# Effects of thiamine on vasopressor requirements in patients with septic shock: a prospective randomized controlled trial

**DOI:** 10.1186/s12871-020-01195-4

**Published:** 2020-11-09

**Authors:** Suttasinee Petsakul, Sunthiti Morakul, Viratch Tangsujaritvijit, Parinya Kunawut, Pongsasit Singhatas, Pitsucha Sanguanwit

**Affiliations:** 1 Department of Critical Care Medicine, Faculty of Medicine, Ramathibodi Hospital, Mahidol University, Ratchathewi, Bangkok, 10400 Thailand; 2 Department of Anesthesiology, Faculty of Medicine, Prince of Songkla University, Hatyai, Songkhla, 90110 Thailand; 3 Department of Anesthesiology, Faculty of Medicine, Ramathibodi Hospital, Mahidol University, Bangkok, Thailand; 4 Piyavate Hospital, Bangkok, Thailand; 5 Department of Internal Medicine, Faculty of Medicine, Ramathibodi Hospital, Mahidol University, Bangkok, Thailand; 6 Department of Surgery, Faculty of Medicine, Ramathibodi Hospital, Mahidol University, Bangkok, Thailand; 7 Department of Emergency Medicine, Ramathibodi Hospital, Mahidol University, Bangkok, Thailand

**Keywords:** Thiamine, Vasopressor requirement, Septic shock

## Abstract

**Background:**

Thiamine, an essential vitamin for aerobic metabolism and glutathione cycling, may decrease the effects of critical illnesses. The objective of this study was to determine whether intravenous thiamine administration can reduce vasopressor requirements in patients with septic shock.

**Methods:**

This study was a prospective randomized double-blind placebo-controlled trial. We included adult patients with septic shock who required a vasopressor within 1–24 h after admission between March 2018 and January 2019 at a tertiary hospital in Thailand. Patients were divided into two groups: those who received 200 mg thiamine or those receiving a placebo every 12 h for 7 days or until hospital discharge. The primary outcome was the number of vasopressor-free days over 7 days. The pre-defined sample size was 31 patients per group, and the study was terminated early due to difficult recruitment.

**Results:**

Sixty-two patients were screened and 50 patients were finally enrolled in the study, 25 in each group. There was no difference in the primary outcome of vasopressor-free days within the 7-day period between the thiamine and placebo groups (mean: 4.9 days (1.9) vs. 4.0 days (2.7), *p* = 0.197, mean difference − 0.9, 95% CI (− 2.9 to 0.5)). However, the reductions in lactate (*p* = 0.024) and in the vasopressor dependency index (*p* = 0.02) at 24 h were greater among subjects who received thiamine repletion vs. the placebo. No statistically significant difference was observed in SOFA scores within 7 days, vasopressor dependency index within 4 days and 7 days, or 28-day mortality.

**Conclusions:**

Thiamine was not associated to a significant reduction in vasopressor-free days over 7-days in comparison to placebo in patients with septic shock. Administration of thiamine could be associated with a reduction in vasopressor dependency index and lactate level within 24 h. The study is limited by early stopping and low sample size.

**Trial registration:**

TCTR, TCTR20180310001. Registered 8 March 2018, http://www.clinicaltrials.in.th/index.php?tp=regtrials&menu=trialsearch&smenu=fulltext&task=search&task2=view1&id=3330.

**Supplementary Information:**

**Supplementary information** accompanies this paper at 10.1186/s12871-020-01195-4.

## Background

Thiamine is a water-soluble vitamin-containing antioxidant. In the aerobic metabolism of cells, thiamine is an essential vitamin, acting as a cofactor of pyruvate dehydrogenase and alpha-ketoglutarate transketolase of the Krebs cycle as well as in the pentose-phosphate shuttle that occurs in mitochondria [1]. An experimental sepsis model study found that thiamine deficiency was associated with greater oxidative stress and inflammatory responses [2]. In addition, thiamine deficiency in rats could produce more reactive oxygen species (ROS) as a consequence of acidosis, with an increase in cell apoptosis [3]. Thiamine is one of the metabolic resuscitators shown to produce nicotinamide adenine dinucleotide phosphate (NADPH) in glutathione cycling, inhibiting ROS and resulting in a decrease in microvascular dysfunction, cellular apoptosis and endothelial dysfunction [4].

Patients with septic shock have high metabolic consumption and have been observed to have many manifestations similar to patients with thiamine deficiency syndrome, including vasodilatation, hypotension, cardiac failure and elevated lactate levels [5]. A retrospective study reported the prevalence of thiamine deficiency in septic shock patients to be approximately 20–70%, and patients who survived had a significantly higher body thiamine status than those who died [6]. One retrospective study showed that early use of intravenous thiamine in patients with septic shock was associated with improved lactate clearance and reduced 28-day mortality [7]. However, a recent observational study found, in a nationwide database investigation, no results that supported an association between an early thiamine administration dose after admission and 28-day mortality [8]. To date, clinical evidence outcomes of thiamine remain inconsistent, and thiamine doses of 400 mg per day appear to be safe in clinical trials and may reduce lactate clearance [9, 10]. We hypothesized that thiamine administration in patients with septic shock would decrease vasopressor requirements and organ failure compared with the corresponding outcomes in patients who did not receive thiamine; this was based on the hypothesized role of thiamine as a metabolic resuscitator.

## Methods

We performed a prospective single-centre randomized double-blind placebo-controlled study to determine whether thiamine administration is associated with improvements in clinical outcomes for patients with septic shock. Patients were enrolled at Ramathibodi Hospital, Mahidol University - a tertiary academic medical centre. The study was approved by The Committee on Human Rights, Related to Research Involving Human Subjects and based on the Declaration of Helsinki, Faculty of Medicine Ramathibodi Hospital, Mahidol University (protocol number: ID 12–60-05). Patients or relatives provided written informed consent prior to enrolment. The trial was registered in the Thai Clinical Trials Registry (TCTR20180310001).

We enrolled consecutive patients between March 2018 and January 2019. We included adult patients (≥18 years) who had suspected infections, showed a Sequential Organ Failure Assessment (SOFA) score ≥ 2, were on a vasopressor or had been administered inotropic drugs for at least 1 h but no more than 24 h, and had a lactate level > 2 mmol/L; these patients were enrolled from emergency departments or the inpatient department unit. We excluded patients based on the following criteria: (1) receipt of thiamine > 100 mg within 24 h before enrolment, (2) thiamine allergy or anaphylaxis, (3) pregnancy, (4) cancer or diseases having a 6-month survival rate ≤ 50% and (5) diagnosed cardiac beriberi, peripheral beriberi, Wernicke-Korsakoff syndrome or re-feeding syndrome.

Patients were randomized via 1:1 block computer-generated randomization with conceal envelope technique to receive either thiamine or a placebo. Patients in the thiamine group received 200 mg of thiamine hydrochloride (vitamin B_1_) in 50 mL of 5% DW every 12 h, with continued infusion for 30 min. Patients in the placebo group received 50 mL of 5% DW every 12 h, with continued infusion for 30 min, for 7 days or until discharge of both groups. The placebo was identical in appearance to thiamine; patients, caregivers and outcome assessors remained blinded throughout the study period. Other septic shock management protocols, such as fluid resuscitation, antibiotics and septic work-ups, followed the Surviving Sepsis Campaign guidelines, 2016 [11].

The primary outcome of the present study was vasopressor-free days over 7 days, defined as the number of days in which patients did not receive vasopressor assistance within 7 days after randomization.

Secondary outcomes included lactate reduction and vasopressor dependency index reduction within 24 h after intravenous administration of thiamine, changes in the vasopressor dependency index from baseline to day 7 (or sooner if the patient was discharged), changes in SOFA scores from baseline to day 7 (or sooner if the patient was discharged) and 28-day mortality. Due to the effects of thiamine possibly being shorter than 7 days, patients would either recover or die. We conducted post hoc analyses of the effects of thiamine on changing SOFA scores, the vasopressor dependency index over 4 days and the difference between SOFA scores on days 1 and 4, as similarly performed in previous studies [7].

Thiamine levels were analysed in plasma via the fluorescence technique, which measured thiamine diphosphate (thiamine pyrophosphate), in which the most important and active form is an intracellular compound, making it the best marker of thiamine nutritional status. Thiamine deficiency, utilizing this technique, was defined as a level less than 70 nmol/L [12].

The vasopressor dependency index was calculated from the inotropic score divided by the mean arterial pressure (inotropic score = (dopamine dose × 1) + (dobutamine dose × 1) + (adrenaline dose × 100) + (noradrenaline dose × 100) + (phenylephrine dose × 100)), and all doses were expressed as mcg/kg/min [13].

After patients were enrolled, all patient demographic data were recorded, and blood was drawn for collection to measure the arterial lactate levels at baseline and arterial lactate levels at 24 h after the first dose. All of the patients’ blood was collected for measurement of their thiamine pyrophosphate levels at baseline before intervention, and parameters of the SOFA scores, including creatinine, total bilirubin, the partial pressure of oxygen (PaO_2_), the platelet count [14] and the vasopressor dependency index, were recorded. The Nutrition Risk in Critically ill (NUTRIC) score, which is a nutritional risk assessment tool developed and validated specifically for ICU patients, was recorded [15]. The Acute Physiology And Chronic Health Evaluation II (APACHE II) score was also recorded [16].

Initial sample sizing was calculated according to the primary endpoint: vasopressor-free day of norepinephrine administration among patients with septic shock from a previous study [17]. To detect a mean 20% reduction duration of the thiamine group, we calculated the sample size with a 2-sided type 1 error of 0.05, with a power of 0.80. From this, we estimated the requirement of 31 patients per group, and the planned period for study was 10 months after first enrolment. We also planned an interim analysis.

A comparison of non-normally continuous data was assessed by the Wilcoxon rank sum test and is reported herein as the median with interquartile range. A comparison of normally continuous data was assessed by Student’s t test and is reported herein as the mean with standard deviations. Categorical variables are presented as percentages and were compared using the chi-square or Fisher exact test, as appropriate. A repeated measurement of the SOFA score and vasopressor dependency index within 7 days was analysed by a linear mixed model, and the worst value was imputed to the variable for patients who died during follow-up. For post hoc analysis, we also analysed the linear mixed model by adjusting the baseline and imputing the worst value to the variable for patients who died during follow-up. Kaplan-Meier curves were created for survival and compared with the log-rank test. Statistical analyses were performed using Stata version 15. All hypothesis tests were significant at a level of *p* < 0.05. The analysis was performed with intention to treat.

## Results

**Fig. 1:**
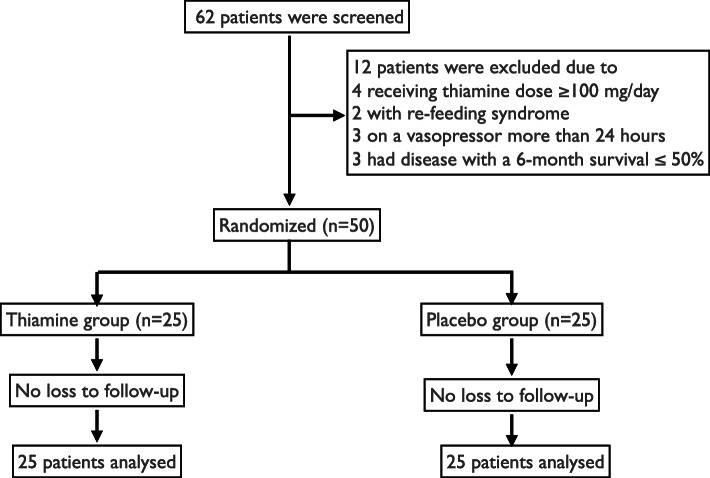
Consort flow diagram

After a total of 10 months, our inclusion criteria were met by 62 patients. From this number, 12 patients were excluded (Fig. 1); the remaining 50 patients were randomized into 2 groups (patient characteristics are shown in Table 1). The predefined sample size was not reached since the number of cases in our hospital was not enough as planned. We found no statistically significant difference in vasopressor free-days between the thiamine group and placebo group (mean of 4.9 days ±1.9 SD in the thiamine group and mean of 4.0 ± 2.7 SD in the placebo group [*p* value: 0.197, mean difference: -0.9, 95% CI: − 2.9 to 0.5]).

**Table 1:** Baseline characteristics of the study patients

Variables	Thiamine (*n* = 25)	Placebo (*n* = 25)
Demographic		
Age, yr, mean (SD)	64 (19.2)	66 (16.7)
Sex, male, n (%)	17 (68)	12 (48)
Weight, mean (SD)	62.5 (20.5)	61.0 (14.6)
BMI, kg/m^2^, mean (SD)	23.3 (6.5)	23.6 (5.7)
Medical ICU, n (%)	16 (64)	22 (88)
Duration of vasopressor treatment, hr., median (IQR)	11 (7–17)	10 (5–12)
Mechanical ventilation and severity of illness		
Mechanical ventilation at the time of enrolment, n (%)	19 (76)	25 (100)
APACHE II score, mean (SD)	26 (7.6)	29 (6.1)
SOFA score at enrolment, mean (SD)	10 (3.9)	11 (2.5)
NUTRIC score, mean (SD)	6 (2.0)	7 (1.7)
Norepinephrine equivalent dose^a^, mcg/kg/min, median (IQR)	0.24 (0.09–0.34)	0.20 (0.07–0.33)
Comorbidities, n (%)		
Diabetes mellitus	12 (48)	15 (60)
Hypertension	14 (56)	20 (80)
Cerebrovascular disease	7 (28)	7 (28)
Coronary artery disease	2 (8)	4 (16)
Chronic heart failure	3 (12)	4 (16)
Chronic obstructive pulmonary disease	0 (0)	0 (0)
Liver disease	5 (20)	4 (16)
ESRD on haemodialysis	1 (4)	4 (16)
Chronic kidney disease	7 (28)	6 (24)
Cancer	9 (36)	11 (44)
Laboratory values at enrolment		
White blood count, × 10^3^, median (IQR)	11.9 (5.7–18.9)	7.8 (1.5–15.2)
Haemoglobin, g/dl, mean (SD)	10.1 (1.9)	10.0 (1.7)
Blood urea nitrogen, mg/dl, median (IQR)	36 (22–58)	43 (20–58)
Creatinine, mg/dl, median (IQR)	1.9 (1.2–2.8)	1.7 (1.3–2.1)
Glucose, mg/dl, mean (SD)	161 (80)	130 (47)
Lactate, mmol/l, median (IQR)	2.9 (2.3–3.5)	2.8 (2.1–5.6)
Thiamine deficiency, n (%)	0 (0)	2 (8)
Thiamine level, median (IQR)	103.9 (77.8–127.0)	86.0 (56.6–124.9)
C-reactive protein, median (IQR)	213.4 (143.6–261.1)	177.1 (81.5–249.9)
Treatment		
Crystalloid, ml, median (IQR)	1500 (800–2500)	1400 (1000–2500)
Colloid, ml, median (IQR)	250 (250–500)	250 (0–500)
Duration of sedation, hr., median (IQR)	1.6 (0–4.0)	2.0 (0–4.0)
Duration of muscle relaxant, hr., median (IQR)	0 (0)	0 (0–1.0)
Hydrocortisone, n (%)	15 (60)	16 (64)
Terlipressin, n (%)	2 (8)	1 (4)
Methylene blue, n (%)	1 (4)	2 (8)
Cytokine removal, n (%)	0 (0)	1 (4)

*n* number, *SD* standard deviation, *hr.* hour, *IQR* interquartile range, *BMI* body mass index, *APACHE II* Acute Physiology and Chronic Health Evaluation, *SOFA* Sequential Organ Failure Assessment, *NUTRIC* Nutrition Risk in the Critically ill, *ESRD* end-stage renal disease

^a^ The norepinephrine equivalent dose was calculated as [norepinephrine (μg/min) + [dopamine (μg/kg/min) ÷ 2] + [epinephrine (μg/min)] + [phenylephrine (μg/min) ÷ 10] [18]

However, there was a statistically significant difference in the vasopressor dependency index, as in a reduction within 24 h in the thiamine group. The median was 0.14 mmHg^− 1^ (IQR: 0.03 to 0.26), which was greater than that in the placebo group, with a median of 0.03 mmHg^− 1^ (IQR: − 0.09 to 0.12), *p* value: 0.020. Moreover, the lactate reduction within 24 h in the thiamine group had a median of 1.0 mmol/L (IQR: − 0.3 to 1.8) and was greatly reduced, more so than in the placebo group: median: 0.5 mmol/L (IQR: − 0.2 to 1.0), *p* value: 0.024 (Table 2).

**Table 2:** Primary outcome and secondary outcomes

Variables	Thiamine (*N* = 25)	Placebo (*N* = 25)	*p*-value
Primary outcome			
No. of vasopressor-free day, mean (SD)	4.9 (1.9)	4 (2.7)	0.197
Secondary outcomes			
24-h lactate reduction, mmol/L, median (IQR)	1.0 (−0.3 to 1.8)	0.5 (−0.2 to 1.0)	0.024*
24-h vasopressor dependency index reduction, mmHg^−1^, median (IQR)	0.14 (0.03 to 0.26)	0.03 (− 0.09 to 0.12)	0.020*
28-day mortality, no./total no. (%)	5 (20)	7 (28)	0.741
SOFA scores day 4 – day 1, median (IQR)	−4 (−5.25 to −1.00)	−4.00 (−6.25 to − 1.50)	0.409

*No.* number, *SD* standard deviation, *IQR* interquartile range

* *p*-value < 0.05

Changes in SOFA scores and the vasopressor dependency index over 7 days are shown in the Supplement. There was no statistically significant difference between the groups.

**Fig. 2:**
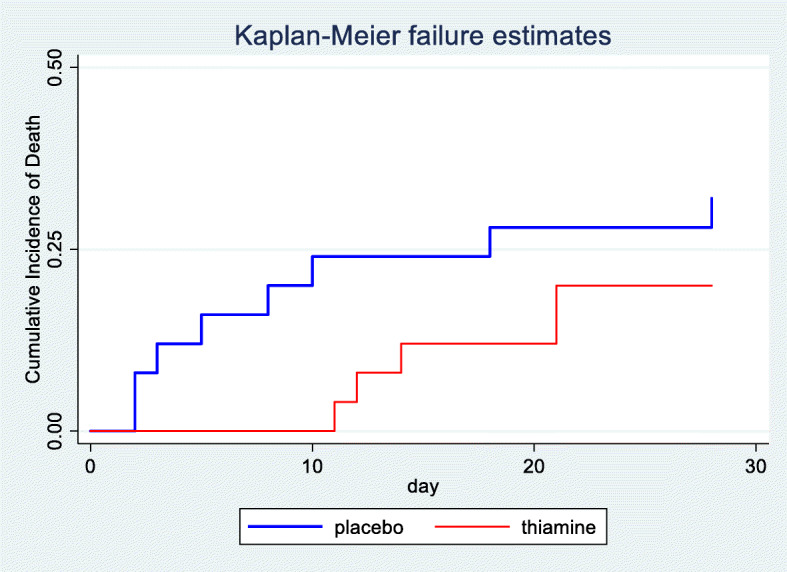
Kaplan-Meier failure estimation 28-day mortality

The 28-day mortality in our study is shown using the Kaplan-Meier failure estimates (Fig. 2), and there was no statistically significant difference (*p* value: 0.395). In the thiamine group, 5 patients died (20%), while in the placebo group, 7 patients died (28%) (*p* value: 0.741) within 28 days. No patients in the thiamine group died within 7 days while receiving thiamine administration; however, 4 patients in the placebo group died over the course of 7 days (Table 2). No adverse effects from thiamine, such as rash, itchy, red skin or anaphylaxis, occurred during the study.

**Fig. 3:**
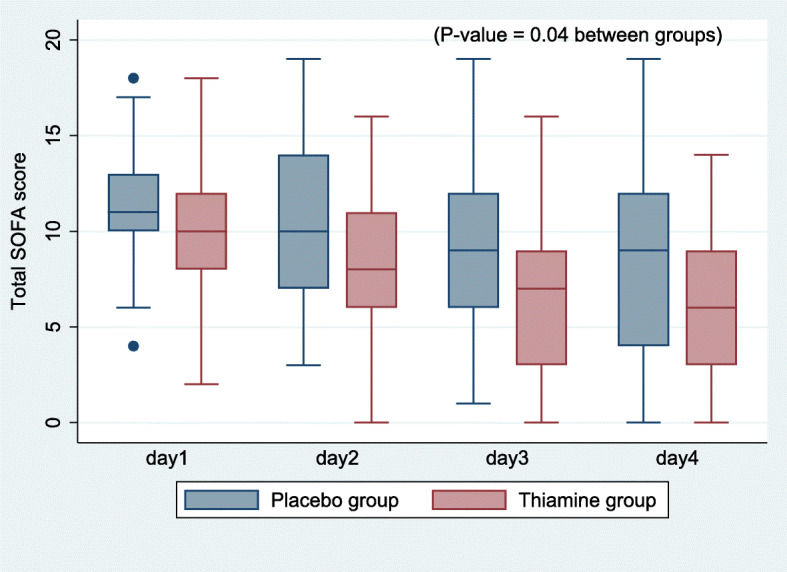
Post hoc exploratory analysis of changes in SOFA scores within 4 days

**Fig. 4:**
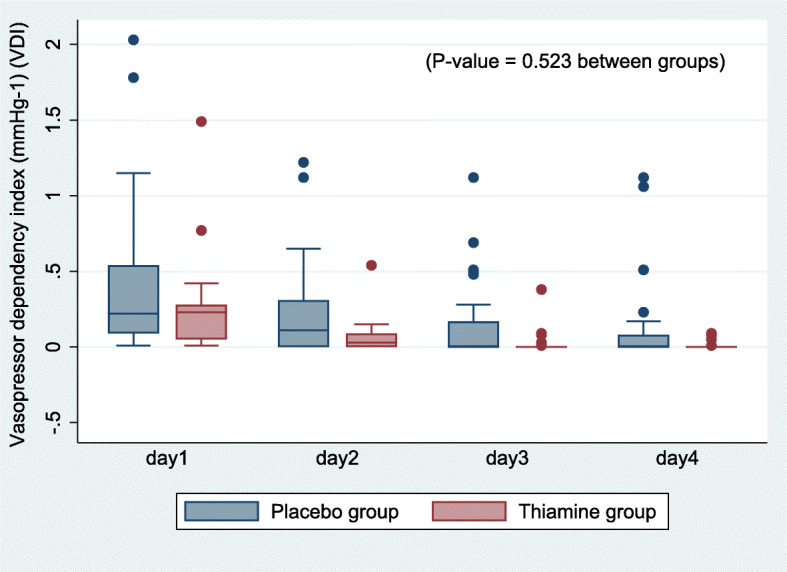
Post hoc exploratory analysis of changes in the vasopressor dependency index within 4 days

For post hoc analysis of the vasopressor dependency index and SOFA scores within 4 days, it was found that changes in SOFA scores within 4 days were significantly different between the groups (p value: 0.04) (Fig. 3); however, changes in the vasopressor dependency index were not significant (p value: 0.523) (Fig. 4).

## Discussion

Taking into account the major limitations of early stopping the study and small sample size, no evidence of differences in vasopressor-free days between the patients receiving thiamine or a placebo in 7 days was found. We found that 24 h after administration of the intervention, patients in the thiamine group had a reduction in their vasopressor dependency index as well as decreased lactate, more so than those in the placebo group. No other secondary outcomes were significantly different.

In general, the rationale for thiamine administration in septic shock is supported by a high incidence of low thiamine levels in critically ill patients and a high thiamine consumption state from increased mitochondrial oxidative stress during critical illnesses [19]. Thiamine plays an important role in producing NADH during glutathione cycling, inhibiting ROS in mitochondria, and therefore may improve microvascular function [4]. From a preliminary study of the thiamine, ascorbic acid, and hydrocortisone drug combination [20] and the post hoc analysis of thiamine supplementation [21], thiamine may prevent organ failure, especially regarding the renal function of patients with septic shock, and may improve global oxygen consumption after CABG surgery [22].

The results of the present study suggest that no difference in the number of vasopressor-free days, mortality, SOFA score or vasopressor dependency index occurred within 7 days. However, within the statistical power limitations, these results also suggest that early administration of thiamine (within 24 h), reduced lactate and the vasopressor dependency index, also within 24 h. Although only a few of the individuals in this study population were thiamine deficient, this number was still less than those other studies. This situation may have been due to the fact that this study differed in population, race, nutritional status and/or early randomization [6, 9]. Larger trials are needed to evaluate the effects of thiamine alone on vasopressor requirements in patients with septic shock.

In our study, we found that thiamine administration could reduce lactate levels compared with those in patients not receiving thiamine. The outcomes were different from previous findings by Donnino et al. [9], in which compared to placebo, thiamine supplementation did not decrease 24-h lactate levels in patients with septic shock and elevated lactate > 3 mmol/L. However, it improved the 24-h lactate clearance in patients with a laboratory-confirmed thiamine deficiency via liquid chromatography/tandem mass spectrometry by Quest Diagnostics, which was different from the findings in our study. Apart from the methods, contrasting outcomes might have occurred in our study due to the limited time dependence of vasopressors (1–24 h) and the earlier enrolment and drug administration than those of other studies [7, 9]. Additionally, our study was different from other investigations, as we used the definition of septic shock taken from the sepsis 3 definition [23]. We included participants with a lactate level > 2 mmol/L.

Regarding physical effects, this study showed a greater reduction in the 24-h vasopressor dependency index in the thiamine group than in the placebo group. We assumed that this improved the microvascular function, as in the pentose-phosphate shuttle that occurs in mitochondria. However, we did not test clinical changes in the cardiac index or vascular resistance. Moreover, we cannot exactly explain the mechanism.

In the post hoc analysis of our study, the outcome of organ failure improved in the first 4 days; therefore, the administration of thiamine might not necessarily last long. On the other hand, the use of thiamine for less than 4 days in the study of Hwang et al. (who administered thiamine and vitamin C to septic shock patients for 2 days) did not improve the SOFA score [24]. However, the administration of thiamine in combination with corticosteroids and ascorbic acid for 4 days in a recent randomized trial likewise did not reduce the SOFA score during the first 72 h [25].

Strengths of our study. First, the design of this study used double blind randomization in that we blinded both the patients and investigators to reduce selection bias. Second, we included patients early and limited the time to randomization. Third, this trial measured thiamine levels at the time of randomization before administering the intervention, which showed a background of thiamine levels in our population with septic shock.

Our study did have limitations. First, it had a small sample size and an early stopping point and was insufficiently powered; this limited validity of the results may have led to selection bias. Moreover, the risk of type I and II errors should be taken into consideration and the results of post hoc analyses should be interpreted with caution. Further studies are needed to evaluate the effectiveness of thiamine on vasopressor requirements. Second, we did not compare differing dosage levels of thiamine. The dosage of thiamine in our study was 200 mg IV every 12 h for 7 days, which was different from that in a study by Woolum JA. et al. [7], in that nearly two-thirds of their thiamine group received high doses of thiamine (500 mg IV) every 8 h for 3 days. In their study, it was found that such levels could decrease mortality. Higher thiamine doses may offer the advantage of improved, passive absorption into the CNS along with improvements in thiamine exposure due to the rapid elimination of thiamine from the serum into urine [26]. Additionally, we did not perform analyses or control the effects of volume resuscitation and other drugs in the septic shock cocktails, consisting of thiamine, hydrocortisone and vitamin C. In our study, more than 60% of the patients from both groups received hydrocortisone, which has potent effects on resolution of shock, and the patient characteristics were severe, similar to the study by Fujii and colleagues in that hydrocortisone might mask the effects of thiamine [27].

## Conclusions

Thiamine was not associated to a significant reduction in vasopressor-free days over 7-days in comparison to placebo in patients with septic shock. Administration of thiamine could be associated with a reduction in vasopressor dependency index and lactate level within 24 h. The study is limited mainly by early stopping and low sample size. Further studies are needed to evaluate the effectiveness of thiamine on vasopressor requirements.

## Supplementary Information

**Additional file 1:** **Figure 1.** Changes of SOFA scores within 7 days. **Figure 2.** Changes of the Vasopressor Dependency index within 7 days.

## Data Availability

The datasets used and/or analysed during this current study are available from the corresponding author, upon reasonable request.

## Abbreviations

ROS: reactive oxygen species
NADPH: nicotinamide adenine dinucleotide phosphate
NADH: nicotinamide adenine dinucleotide
CABG: coronary artery bypass graft
SOFA: Sequential Organ Failure Assessment
PaO2: partial pressure of oxygen
NUTRIC: Nutrition Risk in the Critically ill
ESRD: End-stage renal disease
