# Supplementary material for: Effects of thiamine on vasopressor requirements in patients with septic shock: a prospective randomized controlled trial
Source: BMC Anesthesiol. 2020 Nov 9;20:280. doi: 10.1186/s12871-020-01195-4 (PMC7650202; doi:10.1186/s12871-020-01195-4)

**Supplementary Appendix**

Figure 1. Changes of SOFA scores within 7 days


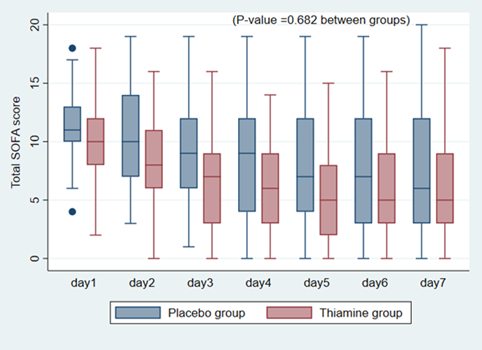


Figure 2. Changes of the Vasopressor Dependency index within 7 days


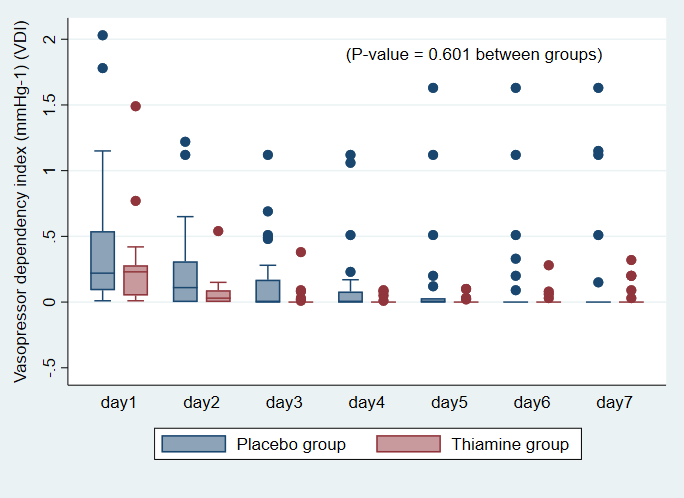

Supplement: Supplementary file 1 — Additional file 1: Figure 1. Changes of SOFA scores within 7 days. Figure 2. Changes of the Vasopressor Dependency index within 7 days. [file 12871_2020_1195_MOESM1_ESM.docx]
